# Visualization of rapid electron precipitation via chorus element wave–particle interactions

**DOI:** 10.1038/s41467-018-07996-z

**Published:** 2019-01-16

**Authors:** Mitsunori Ozaki, Yoshizumi Miyoshi, Kazuo Shiokawa, Keisuke Hosokawa, Shin-ichiro Oyama, Ryuho Kataoka, Yusuke Ebihara, Yasunobu Ogawa, Yoshiya Kasahara, Satoshi Yagitani, Yasumasa Kasaba, Atsushi Kumamoto, Fuminori Tsuchiya, Shoya Matsuda, Yuto Katoh, Mitsuru Hikishima, Satoshi Kurita, Yuichi Otsuka, Robert C. Moore, Yoshimasa Tanaka, Masahito Nosé, Tsutomu Nagatsuma, Nozomu Nishitani, Akira Kadokura, Martin Connors, Takumi Inoue, Ayako Matsuoka, Iku Shinohara

**Affiliations:** 1 Graduate School of Natural Science and Technology, Kanazawa University, Kakuma-machi, Kanazawa, 920-1192 Japan; 2 Institute for Space–Earth Environmental Research, Nagoya University, Furo-cho, Chikusa-ku, Nagoya, 464-8601 Japan; 3 Graduate School of Informatics and Engineering, The University of Electro-Communications, 1-5-1, Chofugaoka, Chofu, 182-8585 Japan; 4 Ionosphere Research Unit, University of Oulu, Oulu, Finland; 5 National Institute of Polar Research, 10-3, Midori-cho, Tachikawa, 190-8518 Japan; 6 The Graduate University for Advanced Studies (SOKENDAI), 10-3, Midori-cho, Tachikawa, 190-8518 Japan; 7 Research Institute for Sustainable Humanosphere, Kyoto University, Gokasho Uji, 611-0011 Japan; 8 Graduate School of Science, Tohoku University, 6-3, Aramaki Aza-Aoba, Aoba-ku, Sendai, 980-8578 Japan; 9 Institute of Space and Astronautical Science, Japan Aerospace Exploration Agency, 3-1-1, Yoshinodai, Chuo-ku, Sagamihara, 252-5210 Japan; 10 Department of Electrical and Computer Engineering, University of Florida, Gainesville, FL 32611-6130 USA; 11 Joint Support-Center for Data Science Research, Research Organization of Information and Systems, 10-3, Midori-cho, Tachikawa, 190-0014 Japan; 12 National Institute of Information and Communications Technology, 4-2-1, Nukui-Kitamachi, Koganei, 184-8795 Japan; 13 Center for Science, Athabasca University, 1 University Drive, Athabasca, AB T9S 3A3 Canada

## Abstract

Chorus waves, among the most intense electromagnetic emissions in the Earth’s magnetosphere, magnetized planets, and laboratory plasmas, play an important role in the acceleration and loss of energetic electrons in the plasma universe through resonant interactions with electrons. However, the spatial evolution of the electron resonant interactions with electromagnetic waves remains poorly understood owing to imaging difficulties. Here we provide a compelling visualization of chorus element wave–particle interactions in the Earth’s magnetosphere. Through in-situ measurements of chorus waveforms with the Arase satellite and transient auroral flashes from electron precipitation events as detected by 100-Hz video sampling from the ground, Earth’s aurora becomes a display for the resonant interactions. Our observations capture an asymmetric spatial development, correlated strongly with the amplitude variation of discrete chorus elements. This finding is not theoretically predicted but helps in understanding the rapid scattering processes of energetic electrons near the Earth and other magnetized planets.

## Abstract (web summary)

Electron precipitation plays major role in magnetospheric physics and space weather. Here the authors show nonlinear behavior of the wave–particle interaction in the magnetosphere as the evolution of chorus electromagnetic waves detected by the Arase satellite and PWING observatory.

## Introduction

Chorus waves are among the most common plasma waves in the Earth’s magnetosphere^1–5^, around magnetized planets^6^, and in laboratory plasmas^7^. Chorus waves in the Earth’s magnetosphere are a major driver of relativistic electron precipitation (MeV or sub-MeV)^8–10^, X-ray microbursts (> 30 keV)^11^, pulsating auroras (tens of keV)^12,13^, and diffuse auroras (keV to tens of keV)^14^, all of which are mediated by chorus wave–particle interactions^15,16^. In particular, coherent chorus elements provide nonlinear energy exchange processes between electromagnetic waves and electrons on microphysical timescales^17,18^, which are essential to rapid local acceleration in the Earth’s radiation belts^19^ and are an important space weather proxy^20^. Theoretical analyses^16,21^ and numerical simulations^22–25^ assuming a one-dimensional spatial model along the background magnetic field line have predicted that rapid resonant interactions occur between single packets of chorus waves and energetic electrons over a wide (keV to MeV) range of energies and at a nonlinear transition time of less than several hundred milliseconds. However, to date there have been no observations that allow the spatiotemporal evolution in the transverse (across an existing geomagnetic field line) direction of single chorus element wave–particle interactions showing an elementary physical step of the prompt resonant interactions, and hence the quantitative spatial development of the source region is still poorly understood. Identifying any trace of the ionospheric footprint along geomagnetic field lines from the location of the chorus source (the magnetic equator)^26^ is difficult because a single chorus-wave packet lasts for only a few hundreds of milliseconds. If a single chorus-wave packet can interact with energetic electrons at the magnetic equator, as suggested by nonlinear theoretical and numerical studies^16,21–25^, a short-lived visible burst should be observed in the magnetic-conjugate region in the auroral zone. To quantify this rapid precipitation of energetic electrons, measurements of the spatiotemporal characteristics of the interaction between chorus waves and electrons are essential. Such electrons affect the Earth’s atmospheric chemistry and may play a role in climate change^27–29^. Direct detection of the auroral flash caused by a single chorus-wave packet requires coordinated observations from a satellite near the source region of the chorus waves at the magnetic equator and from highly sensitive cameras in a ground station network. The Arase satellite^30^ was launched in December 2016 to provide in situ observations in the Earth’s magnetosphere, and the ground-based PWING (study of dynamical variation of Particles and Waves in the INner magnetosphere using Ground-based network observations)^31^ collaboration is underway to deliver unique conjugate observations covering a wide longitudinal range at subauroral latitudes. To deliver unprecedented observations of the short-period auroras associated with chorus-wave packets, ground-based optical observations taken by PWING offer a high temporal resolution at 100 frames per second and the high sensitivity of electron-multiplying charge-coupled device (EMCCD) image sensors.

The spatiotemporal evolution of the auroral flashes driven by discrete chorus elements we show here is consistent with the idea of prompt resonant interactions between electromagnetic waves and electrons in collisionless plasmas. Our observations demonstrate that chorus waves regulate rapidly the time variations and locations of precipitating electron flux into the Earth’s atmosphere. Quantifying the spatial and temporal scales of these precipitation events contributes to understanding the nonlinear physical processes mediating the acceleration and precipitation of energetic electrons on the Earth and other magnetized planets.

## Results

### Coordinated Arase and ground observations

**Fig. 1:**
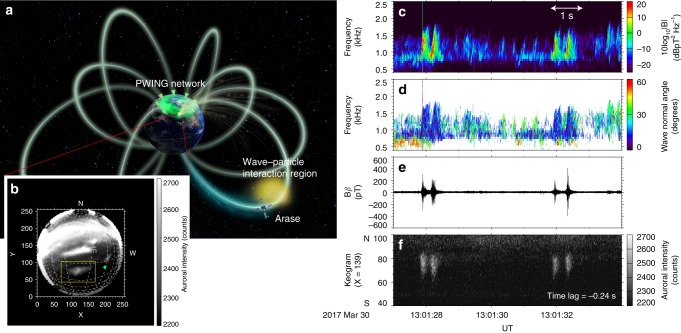
Coordinated PWING stations and Arase observations. **a** Illustration of conjugate observations from the PWING ground network and the Arase satellite. Earth in Fig. 1a is mapped using NASA image. All rights reserved. **b** All-sky EMCCD snapshot observed at Gakona in Alaska at 13:01:28 UT, 30 March 2017. Dotted lines indicate the spacing of geographical latitudes and longitudes at 1° and 2° intervals. The yellow frame indicates the auroral region that is focused on in this study. The green diamond symbol indicates Arase’s footprint at the time of observation. **c**–**e** The dynamic spectrum, wave-normal angle with respect to the geomagnetic field line, and the waveform of large-amplitude chorus elements observed by Arase near the magnetic equator. **f** North to South keogram of auroral emissions with a time shift of −0.24 s

Figure 1a shows an illustration of an observation of a chorus-wave packet taken by the Arase satellite^32–34^, which coincided with an auroral flash that was imaged from the ground at Gakona, Alaska (62.39° N, 214.78° E) at 13:01 UT on 30 March 2017. A time-lapse video of the simultaneously observed all-sky EMCCD image and chorus-wave packets is included in Supplementary Movie 1. At this moment, the Arase satellite was located at the geomagnetic latitude of −19.6° and [*X*_SM_, *Y*_SM_, *Z*_SM_] = [−3.59, −2.15, −1.43] Earth radii (Re) in solar magnetic (SM) coordinates, where the *Z*_SM_-axis is parallel to the Earth’s north magnetic pole, the *Y*_SM_-axis is perpendicular to the Earth–Sun line toward the dusk, and the *X*_SM_*Z*_SM_-plane contains the Earth–Sun direction. The Arase footprint calculated from an empirical geomagnetic field (T02) model^35^ was located near the auroral region (Fig. 1b) associated with the observed chorus-wave packets (Fig. 1c). The individual wave packet propagating parallel to the geomagnetic field line in this event (Fig. 1d) is the large-amplitude chorus (over a few hundreds of pT as shown in Fig. 1e)^36^, which can resonate with not only tens of keV electrons but also relativistic electrons^8–10^ when a resonance condition is satisfied^22^. Additionally, oblique-propagating chorus-wave packets with weak amplitudes were observed simultaneously with a typical pulsating aurora shown at the edge of the yellow frame in Supplementary Movie 1 from 13:01:30 to 13:01:31.5 UT. The equatorial half-gyrofrequency, calculated using the T02 model^35^, is ~2 kHz. The auroral data (Fig. 1f) were time-shifted with a lag of −0.24 s to adjust the temporal coincidence between the discrete chorus elements and auroral flashes. The observed time lag includes information on the source location along the field line.

### Source region

**Fig. 2:**
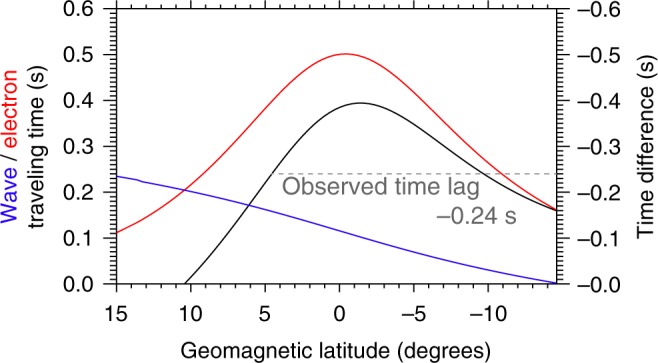
Theoretical time difference between chorus waves and electrons. Blue and red curves indicate the wave and electron traveling times. The dotted line indicates the observed time lag of −0.24 s for reference. Cross points between the black curve and the dotted line indicate the possible location of wave–particle interactions

To localize the source region for chorus wave–particle interactions, the theoretical time difference between a chorus wave with group velocity *v*_g_ and energetic particles with parallel velocity *v*_p_ is calculated as

$$t_{{\mathrm{diff}}} = \mathop {\int }\nolimits_{\hskip 0pt {s_i}}^{s_{{\mathrm{Arase}}}} \frac{{{\mathrm{d}}s}}{{v_{\mathrm{g}}}} - \mathop {\int }\nolimits_{\hskip 0pt {s_i}}^{s_{{\mathrm{ionosphere}}}} \frac{{{\mathrm{d}}s}}{{v_{\mathrm{p}}}},$$

where *s*_*i*_, *s*_Arase_, *s*_ionosphere_, and *s* are the possible source location, the Arase location, the ionospheric location above the ground station (at an altitude of 110 km), and the location along a geomagnetic field line, respectively. The parallel velocity of an energetic electron is written as

$$v_{\mathrm{p}} = \sqrt {\left( {\frac{{2E_{\mathrm{R}}\left( {s_i} \right)}}{m}} \right)\left( {1 - {\mathrm{sin}}^2\alpha \left( {s_i} \right)\frac{{B(s)}}{{B\left( {s_i} \right)}}} \right)} ,$$

where

$$E_{\mathrm{R}}\left( {s_i} \right) = \frac{{B^2\left( {s_i} \right)}}{{2\mu _0N\left( {s_i} \right)}} \cdot \frac{{f_{{\mathrm{ce}}}\left( {s_i} \right)}}{f} \cdot \left( {1 - \frac{f}{{f_{{\mathrm{ce}}}\left( {s_i} \right)}}} \right)^3$$

is the minimum kinetic energy for cyclotron resonance^15^ with chorus waves, *m* is the electron mass, *α* is the pitch angle, *B* is the geomagnetic field, *μ*_0_ is the permeability of a vacuum, *N* is the electron number density, *f* is the wave frequency, and *f*_ce_ is the electron cyclotron frequency. The group velocity^2^ of the chorus wave is

$$v_{\mathrm{g}} = 2c\frac{{f^{1/2}\left( {f_{{\mathrm{ce}}}\left( s \right){\mathrm{cos}}\theta _{{\mathrm{kB}}} - f} \right)^{3/2}}}{{f_{{\mathrm{p}}}\left( s \right)f_{\mathrm{ce}}\left( s \right){\mathrm{cos}}\theta _{{\mathrm{kB}}}}}$$

where, *c* is the speed of light, *f*_p_ is the plasma frequency, and *θ*_kB_ is the wave-normal angle with the geomagnetic field line. The wave-normal angle is assumed to be parallel to the direction of propagation from the possible source location for the sake of simplicity. The electron number density along the geomagnetic field line is assumed to be 1.95 particles cm^−3^, as estimated from the upper hybrid resonance frequency observed by the Arase satellite^37^. The geomagnetic field line is calculated from the T02 model^35^. Figure 2 shows the calculation results for the wave (of 1600 Hz) and the electron traveling time as a function of the geomagnetic latitude of the source location. The observed time lag of −0.24 s is consistent with the theoretical time difference at the geomagnetic latitudes of +4.4° and −9.5°. The source region is localized using a calculation in the observed wave frequency range.

**Fig. 3:**
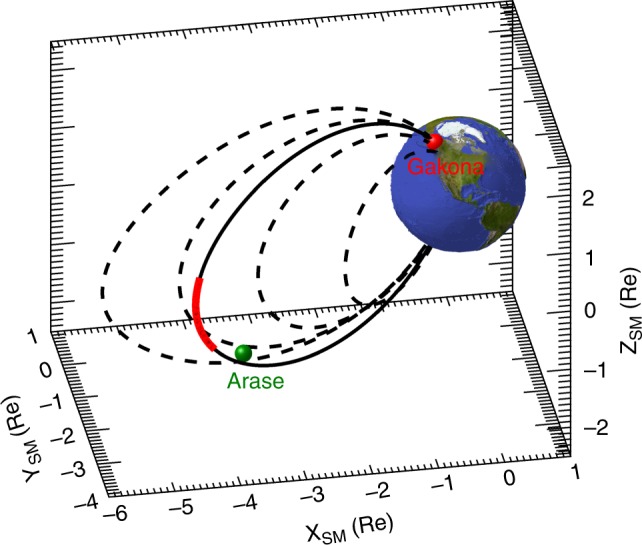
Magnetic latitude range of the wave–particle interaction region. The black solid curve is the magnetic field line connected to the auroral flash observed at Gakona. Dotted curves are magnetic field lines for the L-values of 3, 4, 5, and 6 for reference. The green symbol indicates Arase’s location in SM coordinates

Figure 3 shows the estimated magnetic latitude range of the chorus wave–particle interaction region in SM coordinates. The geomagnetic latitude is within the range +4.4° to −9.5° around the equator. The minimum kinetic energy is in the range from 26 to 83 keV and resonates with chorus waves at frequencies from 1600 to 1150 Hz. When an empirical plasma model, i.e., the global core plasma model^38^, is used instead of the assumption of constant electron density along the geomagnetic field line, the source region was in the range from −3.1° to −12°, still distributing near the equator. Since the time difference between chorus waves and energetic electrons is mostly characterized near the equator as shown in Fig. 2, the inferred source region does not strongly depend on the inhomogeneity of electron density along the geomagnetic field line. Therefore, the observed time difference between chorus elements and auroral flashes suggests that the source of the resonant interactions is located near the magnetic equator, which is in support of early theoretical analyses^39,40^ and numerical simulations^41^ of chorus-wave generation. Because related chorus waves were not detected at the Chistochina ground station, located 40 km northeast of Gakona, the observed chorus waves might be in the so-called nonducted mode in the Earth’s magnetosphere^2^. Nonducted chorus waves can deviate from the geomagnetic field line, thus the Arase satellite, locating a slightly different position from the source region, could observe the chorus elements propagating from the resonant interaction region.

### Spatial development

**Fig. 4:**
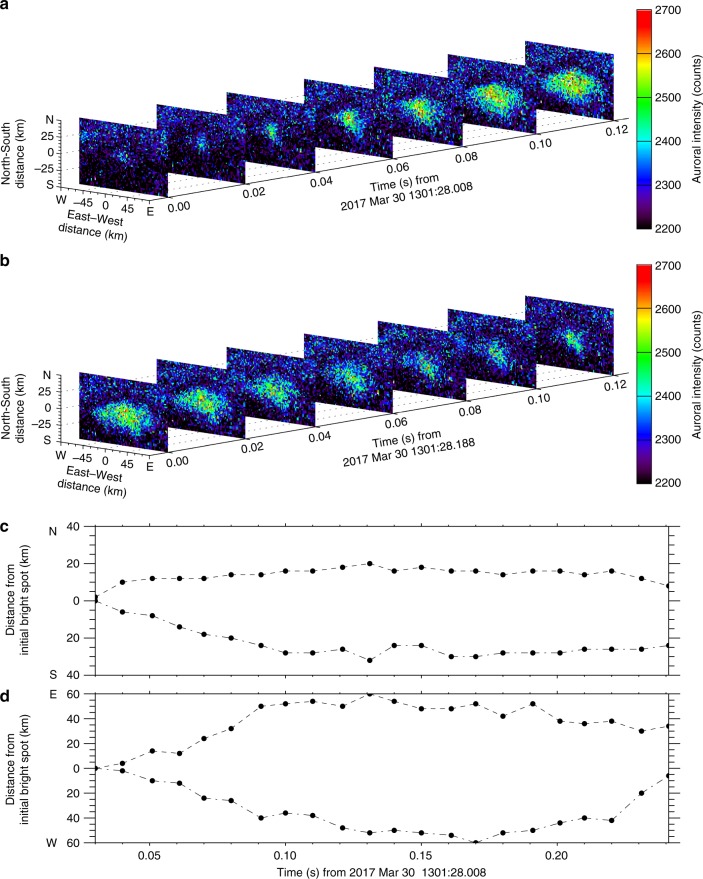
Spatiotemporal evolution of the auroral flash in the ionosphere. **a** The initial spatial expansion and **b** the gradual contraction of the auroral flash. The spatial developments in **c** the magnetic north (dashed line)–south (dot-and-dash line) and **d** east (dashed line)–west (dot-and-dash line) directions from the initial bright spot

The correlated auroral flashes showed rapid spatial expansion (Fig. 4a) and contraction (Fig. 4b) in the ionosphere (at 110 km) as shown in Fig. 4. The auroral flashes expanded and contracted from their original position, which is likely to be the region from which the aurora was seeded. The spatial evolution in the ionosphere shows quite different spatiotemporal characteristics in comparison with a global morphology of diffuse auroras driven by chorus waves in the same manner^14^ and implies that the chorus wave–particle interaction region is also activated in a very small region in the equatorial plane of the magnetosphere. The spatial variations in the magnetic north–south and east–west directions are plotted in Fig. 4c, d. The auroral boundary evolved in the east–west direction with a slight asymmetry but deformed in the magnetic north–south direction with a clear asymmetry. The maximum spatial development in the south direction is approximately two times larger than that for the magnetic north direction from the initial bright spot observed at 1301:28.038 UT. During the growth phase (Fig. 4a), the horizontal expansion speeds were 560 to 625 km s^−1^ in the east–west direction and 250 to 375 km s^−1^ in the magnetic southward direction at the ionospheric altitude (110 km). During the contraction phase (Fig. 4b), the horizontal decay speeds were slower than those in the growth phase. As shown in Supplementary Movie 1, the other auroral flashes also showed similar spatial developments and horizontal speeds. As these speeds are a few hundred times faster than the typical ionospheric convection^42^, the spatial evolution indicates the projection of chorus wave–particle interactions in the magnetosphere without influence from the convection motion. The patchy structure and the expanded development are similar to the spatial features of pulsating aurora^43^, which is also driven by chorus waves consisting of a bundle of successive discrete elements, not a single chorus element. It should be noted that the expansion speeds of the present events were two times or more faster than those of typical pulsating aurora. The rapid development of the coherent spatial structure suggests that the coherent resonant interaction processes along the background magnetic field line between electromagnetic waves and energetic particles are spread radially across the magnetic field line in the seed region near the equator.

**Fig. 5:**
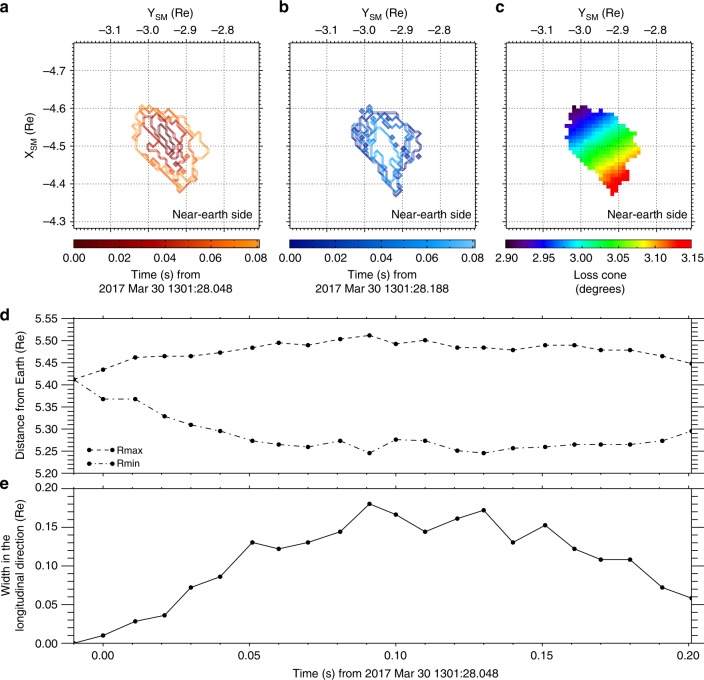
Visualization of the region of resonant interaction between a single chorus wave and electrons. **a** The expansion (red contours) and **b** contraction (blue contours) of the chorus wave–particle interaction region at the magnetic equator (*Z*_SM_ = 0) in SM coordinates. Contour lines are plotted every 20 ms. **c** The spatial dependence of the equatorial loss cone calculated from the T02 model. The time variations of **d** the maximum and minimum radial distance from the Earth and **e** the width in the longitudinal direction of the resonant interaction region

The transverse (across the geomagnetic field line) spatial evolution of chorus wave–particle interactions was estimated by projecting the ionospheric aurora onto the equatorial plane in the magnetosphere using the T02 model^35^. Figure 5a, b shows the spatial evolution of the auroral boundary in the magnetosphere’s equatorial plane. The auroral boundary was extracted using Otsu’s method^44^. The radial distance of the contours mapped along geomagnetic field lines from the Earth to the equatorial plane (*Z*_SM_ = 0) is defined as $R = \sqrt {X_{{\mathrm{SM}}}^2 + Y_{{\mathrm{SM}}}^2}$, and the maximum and minimum values (*R*_max_ and *R*_min_) are plotted in Fig. 5d. The width of contours in the longitudinal direction is plotted in Fig. 5e. The small transverse scale in both radial and longitudinal directions evolves from the order of gyroradii scales of tens-keV electrons at the initial extent phase, then the maximum spatial size is rapidly reached at 0.1 s from the initial development. The maximum widths in the radial and longitudinal directions were 0.25 Re and 0.18 Re, respectively. The observed spatial variation of the resonant interaction in the magnetosphere was mainly the radial expansion toward the Earth rather than in the longitudinal direction. The precipitated electrons must have their pitch angles within an equatorial loss cone, $\alpha _{{\mathrm{LC}}} = {\mathrm{sin}}^{ - 1}\left( {\sqrt {B_0{\mathrm{/}}B_{\mathrm{m}}} } \right)$, where *B*_0_ and *B*_m_ are the geomagnetic field intensity at the magnetic equator and at the ionospheric altitude (110 km), respectively. As shown in Fig. 5c, the angles of this equatorial loss cone, that is, the angle of motion of the electrons relative to the field line within which they must precipitate, as estimated by the T02 model^35^, becomes smaller with increasing radial distance from the Earth. The spatial dependence of the equatorial loss cone can have an impact on this asymmetric distribution in the earthward direction of the resonant wave–particle interaction region, because the earthward loss cone has a wider angle for electron precipitation into the atmosphere.

### Spatiotemporal evolution

**Fig. 6:**
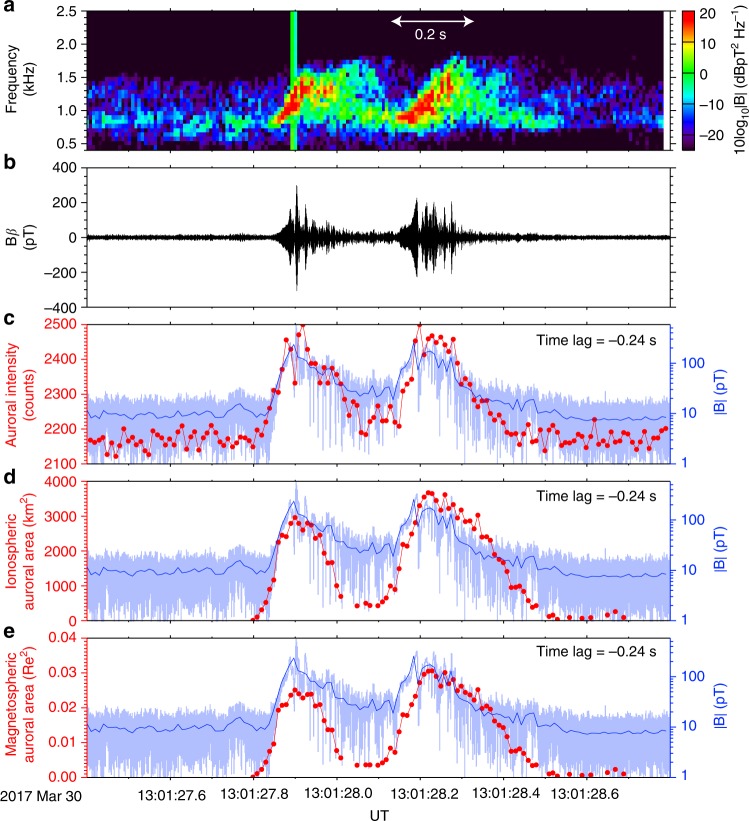
Evolution of chorus element wave–particle interactions associated with the chorus-wave growth. **a,** **b** The frequency–time diagram and the waveform of discrete chorus elements. **c** The temporal evolution of the auroral emissions. Spatial evolutions of the auroral area **d** in the ionosphere (110 km) and **e** at the magnetic equator. The light and rich blue curves indicate chorus amplitudes with a temporal resolution of 1/65536 s and an averaged resolution of 1/100 s in panels **c, d,** and **e**

Figure 6 shows the relationship between the chorus-wave amplitude and the spatiotemporal evolution of the resonant interaction region for the first two chorus elements (Fig. 6a) in Fig. 1c. The chorus-wave amplitude with the original temporal resolution of 1/65536 s (Fig. 6b) is averaged over 0.01 s to match the temporal resolution of the auroral images. The exact one-to-one correspondence between auroral intensity variation and chorus-wave amplitude (Fig. 6c) provides clear experimental evidence that discrete chorus elements effectively and rapidly resonate with energetic electrons at the magnetic equator on the nonlinear scale of a few hundred milliseconds. Furthermore, the expansion and contraction of the auroral area in the ionosphere (Fig. 6d) and at the equatorial plane of the magnetosphere (Fig. 6e) correlates very well with the chorus-wave amplitude, with a correlation coefficient of 0.77. Although there is no information on the transverse size of the chorus-wave packet because of the single probe by Arase, the spatiotemporal evolution of the resonant interaction region in this event showed a clear agreement with the chorus amplitude variation at the Arase location. The relationship between auroral spatial scale and chorus is also reported in the case of typical pulsating aurora, which was accompanied by dense discrete chorus elements^45^. This agreement extracted from the pure single chorus element suggests the possibility that the chorus-wave amplitude is associated with its transverse size of wave packet to effectively and rapidly grow in amplitude. It is known that the chorus-wave amplitude is closely related to various physical parameters, such as the frequency sweep rate, the energy gain of the energetic electrons, propagation effects, and others^17^. The spatial expansion and contraction of auroras has not been theorized clearly because most theories of chorus-wave generation approximate the chorus wave as a plane wave propagating along a field line^17,39,40^. Our findings require a nonlinear theory to explain the relationship between the evolution of the chorus-wave amplitude and the spatial development of the resonant interaction region across the geomagnetic field line, because the source region should be characterized like a natural antenna with a finite spatial scale radiating chorus waves rather than a point source.

## Discussion

In previous studies, chorus wave–particle interactions have been found to show a coincidence in their temporal characteristics^8–13^. The observations presented here show unprecedented one-to-one correspondence between individual chorus elements and auroral flashes on timescales of a few hundreds of milliseconds. Furthermore, our measurements show clear expansion and contraction of the chorus wave–particle interaction region in the Earth’s magnetosphere using visualization through the auroral display. As shown in Fig. 5a, the transverse development implies the cascade of resonant interactions across the geomagnetic field line among different wave numbers and frequencies with electrons and the largest source in the magnetosphere reached a circle of radius ~0.1–0.15 Re. The transverse source size of a single chorus element wave–particle interaction at the magnetic equator was roughly consistent with observations of relativistic electron microburst precipitation events from multiple CubeSats^46^ and with the size of coherent sources of chorus-wave packets^47^. Relativistic MeV and sub-MeV electrons have been reported to precipitate simultaneously with pulsating auroras driven by chorus waves^48^, which suggests that electrons of a wide range of energies can resonate with chorus waves at higher latitudes along the geomagnetic field line^24^. Therefore, the auroral flashes, that are so clearly associated with large-amplitude chorus waves in the present observation, are caused by tens-keV electron precipitation directly exciting auroral emissions and might be accompanied by microbursts of relativistic electrons when the resonant condition is established^22,48^.

The high correlation between aurora size and chorus amplitude in the observations suggests the following three interpretations: (1) the auroral size indicates the wave–particle interaction region as suggested by previous work^49^ and also indicates the finite transverse size of the chorus-wave source^47^; (2) the area of the source region depends on chorus-wave propagation^49^, which is essential for wave growth^17^, and (3) the auroral size is restricted by the spatial structure of the electron populations that are suited for chorus-wave generation^50^. Further theoretical consideration will be needed to understand the parameters that dominate the spatial evolution of the wave–particle interaction regions.

Chorus waves have attracted considerable attention not only as loss mechanisms of the Earth’s radiation belt electrons but also for their local acceleration via wave–particle interactions^19,51,52^. The localized acceleration and loss rates of energetic electrons from the radiation belts are strongly restricted by the spatial scales of the chorus waves. The visualization of the wave–particle interaction region as an aurora provides a key informational tool for understanding the dynamical variations of energetic electron populations of the Earth’s magnetosphere and other magnetized planets.

## Methods

### Chorus-wave measurements

Chorus waves were observed by the Magnetic Search Coil^34^ of the Plasma Wave Experiment (PWE)^32^ aboard the Arase satellite^30^. The waveforms were captured with a sampling frequency of 65.536 kHz^33^. The wave amplitude $\left| B \right| = \sqrt {B_\alpha ^2 + B_\beta ^2 + B_\gamma ^2}$ was calculated as the absolute value of the magnetic field vector of a chorus wave, where *B*_*γ*_ is a spin axis component of a magnetic field vector and, *B*_*α*_ and *B*_*β*_ are spin-plane components. The wave-normal angle is calculated using Means’ method^53^.

### Ground-based optical observations

Prompt auroral emissions at the first positive bands of molecular nitrogen (~700–900 nm) were recorded at 100 frames per second. EMCCD images were taken with an electron-multiplying (EM) gain of 150 and 2 × 2 binning (total: 256 × 256 pixels). The EMCCD images were triggered by 1-pulse-per-second (1PPS) signals from a global positioning system (GPS) receiver to maintain time synchronization accurate to less than 1 ms. The EMCCD images were taken through an RG665 glass filter to eliminate slow auroral emissions^54^ (i.e., 557.7 and 630.0 nm lines). The high-speed image data allows direct comparisons between chorus-wave packets and short auroral emissions.

### Auroral image mapping

The World Geodetic System 1984 was used for ionosphere mapping of the all-sky EMCCD images. The altitude of the auroral emissions was assumed to be 110 km, and the magnetic declination was assumed to be 18° from the international geomagnetic reference field^55^. While mapping at the magnetosphere, the T02 model^35^ was used with SM coordinates. The auroral contour is extracted from the auroral images mapping in the ionosphere (110 km) using Otsu’s method^44^, then the contour is projected to the equatorial plane using the T02 model^35^. The auroral image at an altitude of 110 km and at the magnetic equator has a spatial resolution of 2 km and 0.01 Earth radii, respectively.

## Supplementary information

Description of Additional Supplementary Files

Supplementary Movie 1

## Data Availability

The EMCCD image by PWING and chorus wave by Arase data that support the findings of this study are available from the ERG Science Center operated by ISAS/JAXA and ISEE/Nagoya University (https://ergsc.isee.nagoya-u.ac.jp/data_info/index.shtml.en).
