# Supplementary material for: Visualization of rapid electron precipitation via chorus element wave–particle interactions
Source: Nat Commun. 2019 Jan 16;10:257. doi: 10.1038/s41467-018-07996-z (PMC6335576; doi:10.1038/s41467-018-07996-z)
Supplement: Supplementary file 1 — Description of Additional Supplementary Files [file 41467_2018_7996_MOESM1_ESM.pdf]

Description of Additional Supplementary Files

File Name: Supplementary Movie 1
Description: Slow-motion movie (7-s duration) of all-sky EMCCD images and simultaneous observed chorus elements at 13:01 UT, 30 March 2017. The auroral emissions in the target area are associated with large-amplitude chorus waves. MPEG (.MP4) movie shows the dynamic spatial evolution of auroral emissions associated with large-amplitude chorus waves. The aurora images were taken at 100 frames per second using a HAMAMATSU EMCCD camera equipped with an RG665 glass filter. The moving bar in the chorus waves traces the time progress of the movie.
